# Band structure analysis of the magneto-optical effect in bcc Fe

**DOI:** 10.1038/s41598-021-00478-1

**Published:** 2021-10-25

**Authors:** Ondřej Stejskal, Martin Veis, Jaroslav Hamrle

**Affiliations:** Faculty of Mathematics and Physics, Charles University, Prague, Czech Republic

**Keywords:** Ferromagnetism, Magneto-optics

## Abstract

Magneto-optical effects are among the basic tools for characterization of magnetic materials. Although these effects are routinely calculated by the ab initio codes, there is very little knowledge about their origin in the electronic structure. Here, we analyze the magneto-optical effect in bcc Fe and show that it originates in avoided band-crossings due to the spin-orbit interaction. Therefore, only limited number of bands and *k*-points in the Brillouin zone contribute to the effect. Furthermore, these contributions always come in pairs with opposite sign but they do not cancel out due to different band curvatures providing different number of contributing reciprocal points. The magneto-optical transitions are classified by the dimensionality of the manifold that is formed by the hybridization of the generating bands as one- or two-dimensional, and by the position relative to the magnetization direction as parallel and perpendicular. The strongest magneto-optical signal is provided by two-dimensional parallel transitions.

## Introduction

The magneto-optical (MO) effects are routinely used for characterization of ferro-, ferri-, and antiferromagnetic materials. Magneto-optics can be employed as spectroscopy tool^1–4^, microscopy tool^5–8^, tool to investigate ultrafast magnetization dynamics^9–12^, etc. The MO effects are used in many applications, such as holographic displays^13–15^, optical isolators^16–18^ and photonic crystals^19–21^.

In ferromagnets, the MO spectra are the result of simultaneous presence of the exchange and spin-orbit (SO) interactions and several theoretical models have been presented in the past^22,23^. The first evaluation of magneto-optical spectrum of iron from first principles was performed by Singh et al.^24^ followed by Oppeneer et al.^25^ evaluating the Kerr rotation spectra of bcc Fe. The rise of the ab initio codes in recent decades have enabled to model the MO spectra for real materials^26,27^ and it has quickly become a common practice in research^28–33^. The ab initio codes also opened up new possibilities in the investigations of the microscopic origins of the MO effect that go beyond the semiclassical models^34,35^. They enable thorough *k*-resolved band-by-band analysis of the MO spectra that can significantly contribute to the understandning of the underlying mechanisms. The importance of a complete band structure analysis is emphasized by recent discoveries of large MO Kerr effect in antiferromagnets potentially connecting MO to Berry curvature effects^36–38^.

In this manuscript, we thoroughly analyze the linear MO effect of bcc Fe and identify the origin of the MO signal in the band structure. We pinpoint the conditions and necessary features of the electronic structure leading to the MO response. Understanding of these features can lead to the enhancement and tuning of the various MO effects (e.g. Kerr effect, Faraday effect, magnetic circular dichroism) with the band structure engineering. The MO transitions are classified by their dimensionality and position in the Brillouin zone with respect to magnetization with each class manifesting differently in the spectra.

## Ab initio description

The electronic structure is calculated by the WIEN2k code^39,40^ with the bcc Fe lattice constant of 2.8665 Å^41^, 729,000 *k*-points in the full Brillouin zone, and the local spin density approximation^42^. The calculation is performed with the magnetization in the *z*-direction and with the spin-orbit interaction. The product of the smallest atomic sphere and the largest reciprocal space vector was set to $R_{\mathrm{MT}}K_{\mathrm{max}}=8$ with the maximum value of the partial waves inside the spheres $l_{\mathrm{max}}=10$. States up to 3*s* are treated as core states. Bands are labeled by an increasing energy eigenvalue starting with 3*p* states.

The linear MO response is described by the off-diagonal element of the permittivity tensor calculated by^43,44^ (SI units):

1 $$\begin{aligned} {\text {Re}}(\varepsilon _{xy}(E))=-\frac{\pi e^2\hbar ^2}{(2\pi )^3m^2\varepsilon _0 E^2} \sum _{i} \sum _{f} \iint \mathrm {d}S \frac{{\text {Im}}[\langle i|p_x|f\rangle \langle f|p_y|i\rangle ]}{|\nabla _{\mathbf {k}}E_{fi}|_{E_{fi}=E}} \end{aligned}$$

The imaginary part of the permittivity tensor is obtained by the Kramers–Kronig relations. The summations run over occupied (labeled *i* as initial states) and unoccupied states (*f* standing for final). $E_{fi}=E_f-E_i$ is the energy difference between pair of bands. The integration runs over a surface of constant energy difference, $E_{fi}(\mathbf {k})=E$, with *E* corresponding to the photon energy. The gradient is evaluated on the surface of constant energy difference.

**Figure 1:**
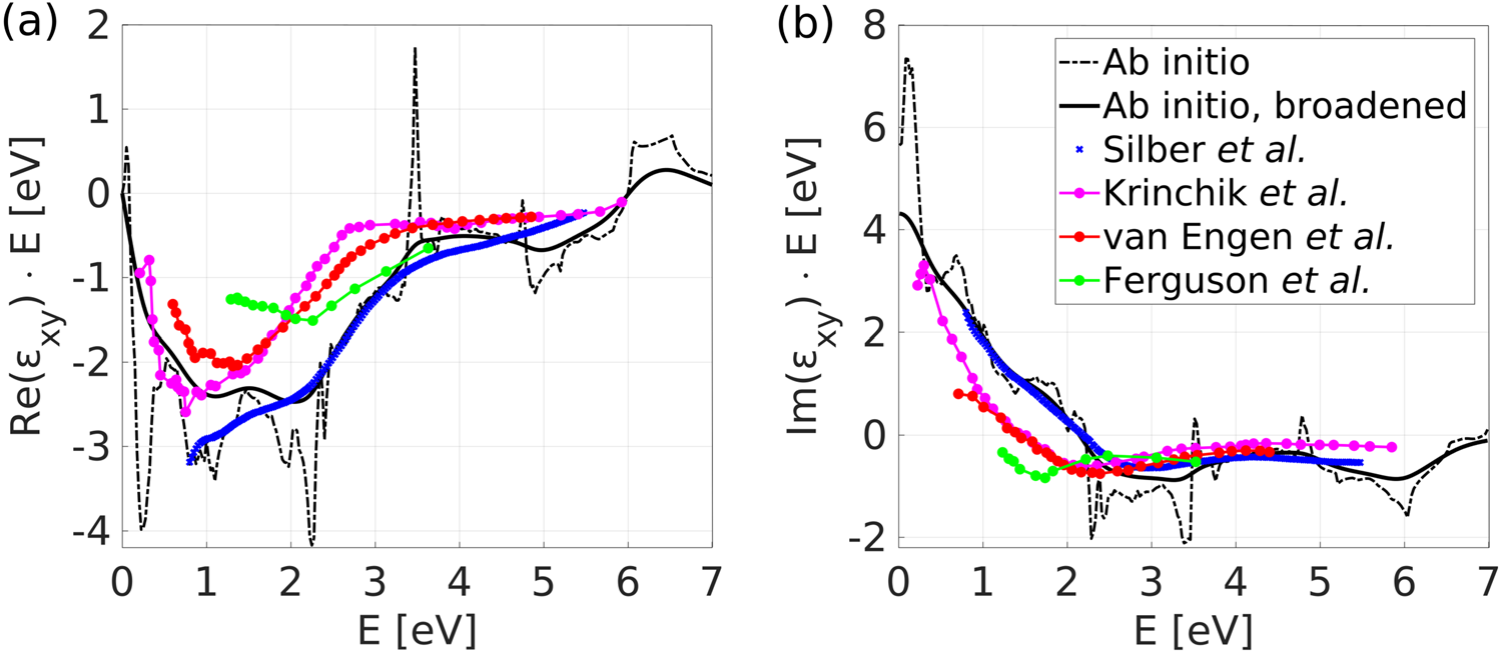
(**a**) Real and (**b**) imaginary part of the linear MO spectra. Black dash-dotted (solid) line is the model without (with) broadening with $\gamma$ = 0.5 eV, respectively, with the imaginary part obtained by the Kramers–Kronig relations. The experimental results are taken from the literature^1,2,25,32^. Two main peaks at 1.1 eV and 2.0 eV in the real part are well described by the model. The 0 eV limit of the imaginary part corresponds to the anomalous Hall conductivity, for unbroadened spectra $\sigma _{xy}^{\mathrm {AHC}}=759.90$ ($\Omega$cm)$^{-1}$ in agreement with previous calculations^45^.

**Figure 2:**
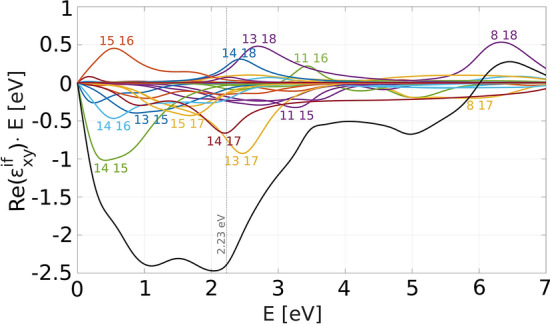
Individual contributions to the real part of the off-diagonal permitivity. The numbers denote the initial and final band indices. Black line represents the total.

**Figure 3:**
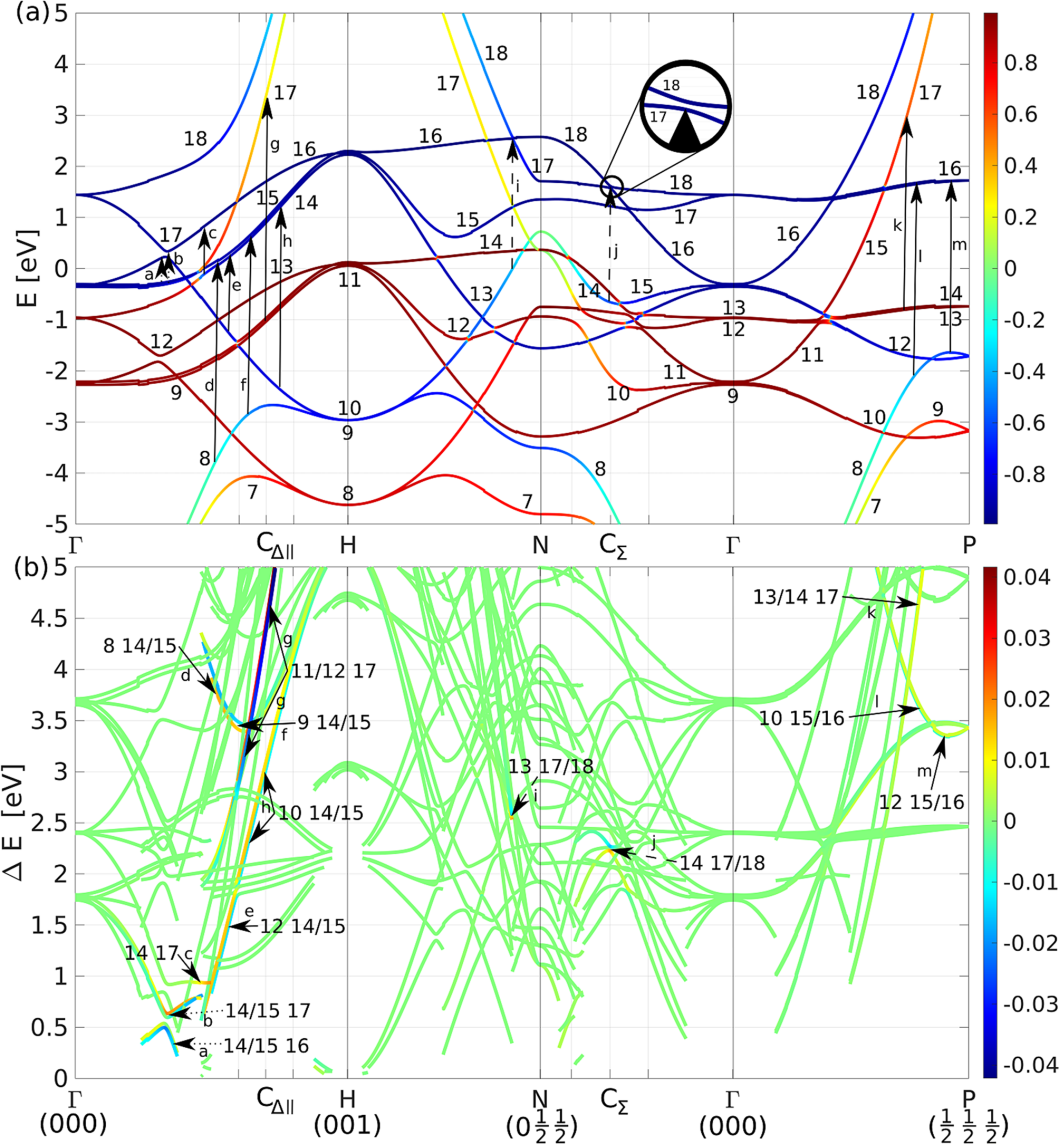
(**a**) Band structure with colors corresponding to the *d*-character of bands with spin-up being positive and spin-down negative. Arrows indicate individual magneto-optical transitions. Fermi level is at 0 eV. (**b**) Joint band structure with colors representing the imaginary part of the product of momentum matrix elements for respective pair of bands ${\text {Im}}[\langle i|p_x|f\rangle \langle f|p_y|i\rangle ]$. Strong transitions are labeled by their respective band indices in correspondence with (**a**). Since they come in pairs, three bands are generally involved in a single transition. Strong 2D MO transitions are marked by dashed arrows and 1D transitions by solid arrows. Transitions a and b are marked by dotted lines as special case. The initial bands involved in these transitions are 1D-split while the final bands are 2D-split. SO splitting of bands 17 and 18 at C$_\Sigma$ is zoomed in.

## Off-diagonal permittivity spectra

Figure 1 shows the calculated MO spectra (off-diagonal permittivity) of bcc Fe compared to experiments^1,2,25,32^. The dash-dotted black line is the direct result of evaluating Eq. (1) and the solid black line is obtained by Lorentzian broadening with $\gamma =0.5$ eV which accounts for finite lifetimes of the excited states and finite temperature and allows for comparison with experiment. The permittivity is multiplied by energy and acquires the units of eV. Therefore, it resembles the conductivity spectrum due to the relation $\varepsilon _{xy}=\frac{i\hbar }{\varepsilon _0 E}\sigma _{xy}$ in SI units. The agreement of the model with all experiments is solid in the entire energy interval.

In order to study the microscopic origin of the MO transitions, we define individual contributions $\varepsilon ^{if}_{xy}$:

2 $$\begin{aligned} \varepsilon _{xy}(E)=\sum _{i} \sum _{f} \varepsilon ^{if}_{xy}(E). \end{aligned}$$

depicted in Fig. 2. Note that there are only several significant contributions.

In Fig. 3a the conventional band structure of bcc Fe is shown. Next, we define the joint band structure (Fig. 3b) that shows the energy difference of all pairs of bands over the same *k*-path with the condition that the upper band lies above the Fermi level and the lower band below it. The colors correspond to ${\text {Im}}[\langle i|p_x|f\rangle \langle f|p_y|i\rangle ]$ in Rydberg units for the respective pair of bands. Green color represents zero, thus no MO signal from the respective pair of bands in the respective *k*-point. Red (blue) color corresponds to the positive (negative) product of the momentum matrix elements and contributes negatively (positively) to the MO spectra due to the minus sign in Eq. (1). Strong transitions are labeled in the joint band structure and located in the conventional band structure.

**Figure 4:**
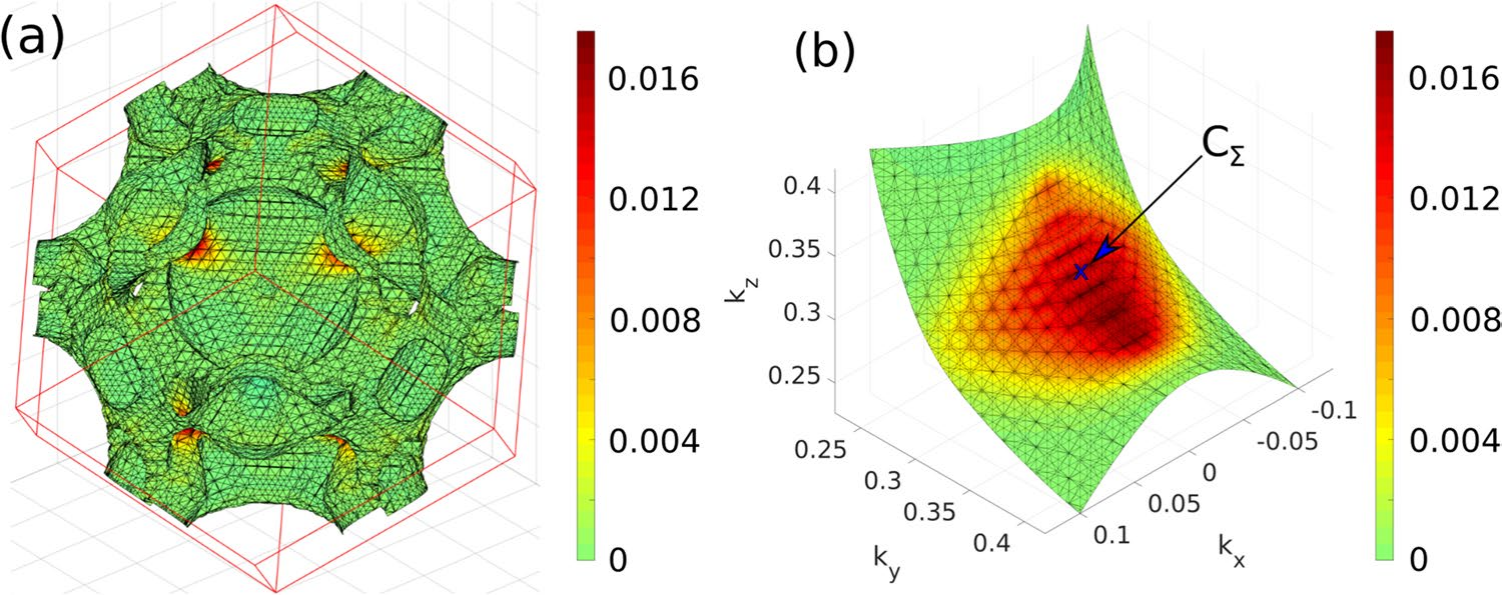
(**a**) Surface of constant energy difference of 2.23 eV between bands 14 and 17 in the Brillouin zone and (**b**) surface of maximal hybridization of bands 17 and 18 in the vicinity of point C$_\Sigma$ with colors corresponding to ${\text {Im}}[\langle 14|p_x|17\rangle \langle 17|p_y|14\rangle ]$ in Rydberg units.

Particular transition can be visualised directly in the Brillouin zone. The contribution to the permittivity is governed by ${\text {Im}}[\langle i|p_x|f\rangle \langle f|p_y|i\rangle ]$ which is integrated over a surface that is unique for a given pair of bands and a given energy (see Eq. (1)). As an example, we present this surface for transition 14 $\rightarrow$ 17 at 2.23 eV in Fig. 4a, which corresponds to the maximal contribution to the permittivity tensor provided by this pair as shown in Fig. 2. This transition originates in a single point in the Brillouin zone denoted as C$_\Sigma =(0, 0.319, 0.319)$ in the $\Gamma$-N direction and is repeated eight times due to symmetry. This transition is labeled j in Fig. 3.

**Figure 5:**
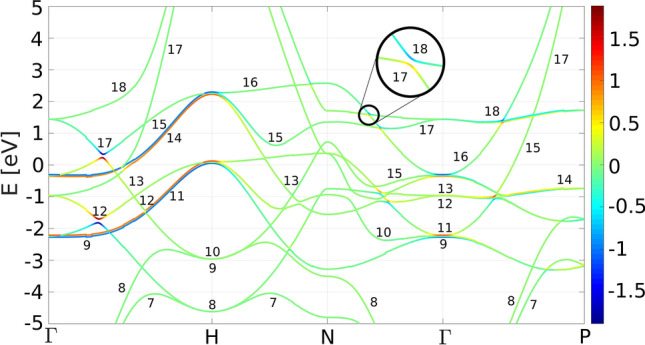
Band structure with colors corresponding to the expectation value of the magnetic number of the *d*-states. The magnetic number gets polarized whenever bands hybridize. The hybridization of bands 17 and 18 at C$_\Sigma$ is zoomed in.

## Magnetic quantum number

Electric dipole transitions are allowed only when certain conditions are met. Apart from the energy and momentum conservation (indirect transitions are not considered), the selection rules dictate the change of the angular momentum, $\Delta j=\pm 1$. This can be observed in Fig. 3a as for every transition either initial or final band loses its almost pure *d*-character (the color gets lighter). The change in the magnetic quantum number, $\Delta m=\pm 1$, then corresponds to the absorption of right or left circularly polarized light, their difference is the MO signal.

In Fig. 5, the band structure is shown with colors corresponding to the expectation value of the magnetic quantum number of the *d*-states. The magnetic quantum number gets polarized when bands approach each other and hybridize. An isolated band will always have its expectation magnetic number equal to zero. This comes from the perfect balance of substates with quantum numbers $+m$ and $-m$ for every real orbital. This balance is broken when two or more orbitals are mixed by the SO interaction^46^.

All strong transitions originate in *k*-points where two *d*-bands energetically approach each other and hybridize by the SO interaction while simultaneously there is a suitable third band with partial *p*-character to enable the transition. Therefore, the strong MO transitions come from several isolated *k*-points in the Brillouin zone. These transitions also come in pairs with opposite signs and slight energy shift and thus tend to cancel each other out as observed in Fig. 2.

## Why the transitions do not cancel?

Transitions come in pairs with similar MO strength and opposite sign, but the spectrum is dominated by negative contributions. This is caused by the joint density of states (JDOS) that in the case of bcc Fe is generally larger for negative contributions. The JDOS can not be shown directly as it by definition also includes states that are optically forbidden.

**Figure 6:**
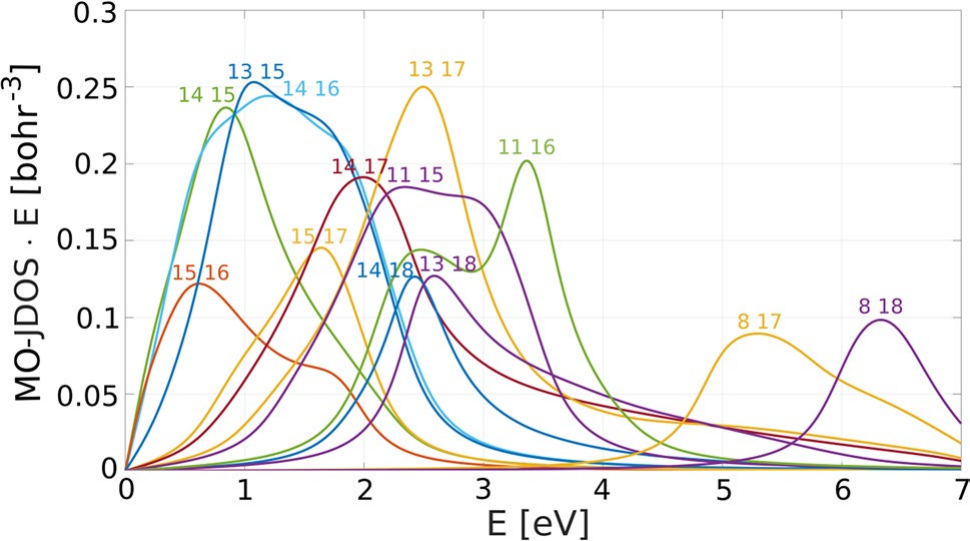
Selected transitions of MO-JDOS multiplied by energy. The numbers denote the initial and final bands. Transitions contributing negatively to the spectrum tend to have larger JDOS.

Therefore, we define the MO-JDOS:

3 $$\begin{aligned} \begin{aligned} \mathrm {MO -JDOS}^{if}(E)=&\frac{1}{(2\pi )^3} \iint \mathrm {d}S \frac{q}{|\nabla _{\mathbf {k}}E_{fi}|_{E_{fi}=E}}, \quad \text {where} \\ q=&{\left\{ \begin{array}{ll} 1 &{} \text {if} \,\, \left| {\text {Im}}[\langle i|p_x|f\rangle \langle f|p_y|i\rangle ]\right| >10^{-4} \\ 0 &{} \text {otherwise} \end{array}\right. } \end{aligned} \end{aligned}$$

which corresponds to the number of *k*-points contributing to the MO transitions. In Fig. 6 only pairs of bands with large MO activity are shown. Note that negatively contributing transitions 13 $\rightarrow$ 17 and 14 $\rightarrow$ 17 have larger MO-JDOS than their positively contributing counterparts 13 $\rightarrow$ 18 and 14 $\rightarrow$ 18. Thus, transitions that contribute negatively to the MO spectrum have larger MO-JDOS most of the time resulting in overall negative spectrum of bcc Fe.

**Figure 7:**
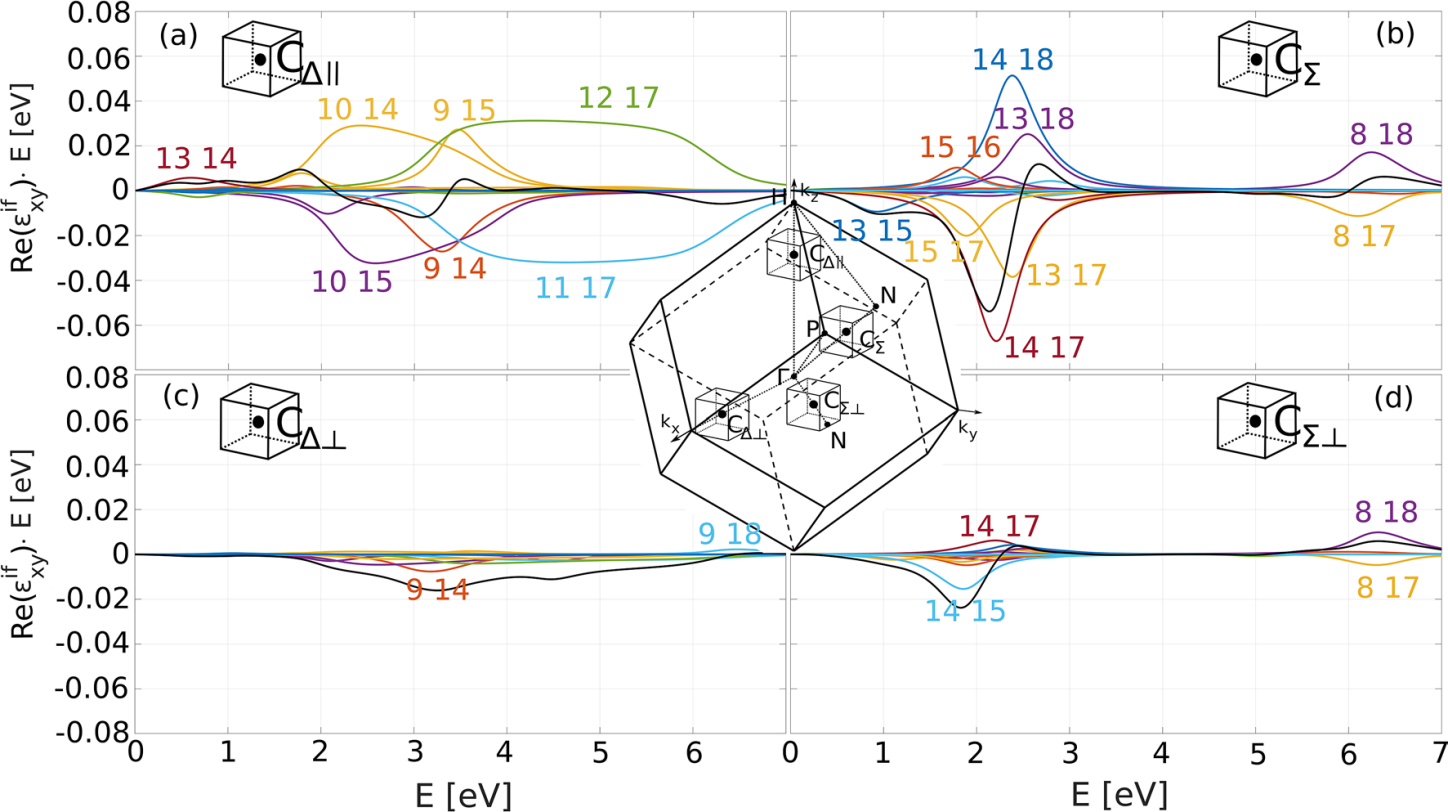
Local MO spectra in equally-sized cubes centered at (**a**) C$_{\Delta \parallel }$, (**b**) C$_{\Sigma }$, (**c**) C$_{\Delta \perp }$, and (**d**) C$_{\Sigma \perp }$ demonstrating the classification of the MO transitions. Magnetization is in the *z*-direction. Black lines represent the totals. In (**a**) the contributions are dominated by 1D transitions that are spread in energy and cancel out, while in (**b**) the MO spectrum is dominated by 2D transitions that exhibit as opposite peaks with an energy offset and the cancellation is only partial. Furthermore, both (**a**) and (**b**) are classified as parallel contributions displaying much larger MO response than perpendicular contributions shown in (**c**) and (**d**).

**Figure 8:**
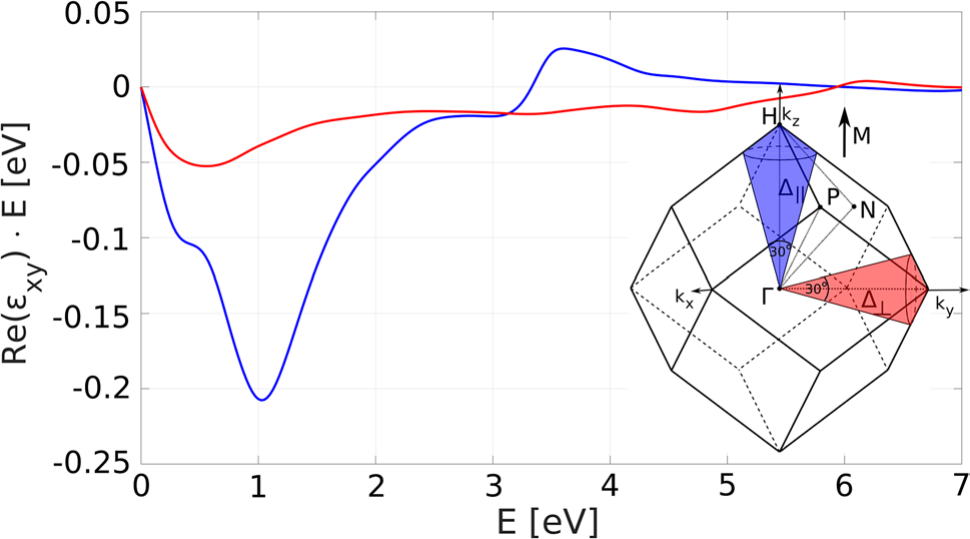
Local MO spectra in cones parallel (blue) and perpendicular (red) to the magnetization M with main axes $\Delta _\parallel$ and $\Delta _\perp$, respectively. The parallel contributions dominate in the entire spectral range.

## Classification of magneto-optical transitions

### Dimensionality of the generating manifold

When two bands approach each other energetically, they hybridize due to the SO interaction. This hybridization polarizes magnetic quantum number and generates MO transitions. The hybridization of the generating bands can be tracked and used to localize *k*-points where maximal hybridization occurs. The set of such *k*-points forms locally a manifold which can be one-dimensional (1D) or two-dimensional (2D). The dimension of this manifold defines the dimensionality of the MO transitions involving these generating bands.

*1D transitions* When touching of two bands is split by the SO interaction, a suitable third band can induce 1D MO transitions into the split touching. An example would be transitions 11 $\rightarrow$ 17 and 12 $\rightarrow$ 17 labelled g in Fig. 3 that originate in hybridization of bands 11 and 12. In this case, both bands hybridize along $\Gamma$-H and diverge in all other directions. The line segment $\Gamma$-H is the 1D manifold generating this transition.

In Fig. 7a we present local MO spectra in the vicinity of C$_{\Delta \parallel }=(0, 0, 0.7)$. It is a result of integration of the Kubo formula (Eq. (1)) in a cubic part of the Brillouin zone centered at C$_{\Delta \parallel }$ with side length of 0.2 (sketched in the inset). The MO response is dominated by 1D transitions that originate from splitting of bands 11/12 and 14/15. Such transitions are typically weak in intensity and spread out in energy. In pairs, they tend to cancel out almost identically, therefore their contribution to the MO spectra is negligible. These transitions are marked by solid arrows in Fig. 3.

*2D transitions* When crossing of two bands is split by the SO interaction, a suitable third band can induce 2D MO transitions into the split crossing. An example would be transitions 14 $\rightarrow$ 17 and 14 $\rightarrow$ 18 labeled j in Fig. 3 that originate in the avoided crossing of bands 17 and 18 at C$_\Sigma$. The surface generating this transition is shown in Fig. 4b and it is a collection of *k*-points where bands 17 and 18 maximally hybridize.

In Fig. 7b local MO spectrum in a cube centered at C$_\Sigma$ is shown. In this part of the Brillouin zone, the MO response is dominated by 2D transitions originating in the splitting of bands 17/18 (and also of bands 16/17 which is also located in this cube as shown in Fig. 3; the vertical lines around point C$_\Sigma$ indicate the edges of the cube). This cube is repeated eight times in the full Brillouin zone, therefore the contribution to the total spectrum coming from this part of the Brillouin zone will be eight times larger which practically covers all the strong peaks around 2–3 eV of the total spectrum. A typical 2D transition consists of two peaks in the MO spectra that are approximately equal in magnitude, have opposite signs and are shifted in energy preventing them from canceling each other. Such transitions exhibit large contributions to the MO spectra and are localized in energy. These transitions are marked by dashed arrows in Fig. 3.

### Position relative to the magnetization

The magnetization vector assumed in the *z*-direction lowers the symmetry of the cubic crystal by breaking the time-reversal symmetry. The band structure therefore slightly differs in directions parallel and perpendicular to the magnetization. Even such minor change can alter the MO contributions significantly.

The difference of the parallel and perpendicular contributions to the total MO spectrum is demonstrated in Figs. 7 and 8 with the parallel ones dominating in the entire spectral range. Bands that generate the transitions are split by SO and hybridize in the parallel direction while they remain degenerate in the perpendicular direction and are magneto-optically inactive.

## Conclusion

The origin of the magneto-optical effect in bcc Fe was thoroughly studied. The MO response originates in avoided band-crossings due to the SO interaction, therefore MO transitions come in pairs with opposite signs and tend to cancel each other. The extent of the cancellation is predominantly determined by the JDOS of the opposite transitions. In the case of Fe, JDOS is larger for negatively contributing transitions, resulting in overall negative spectrum.

**Figure 9:**
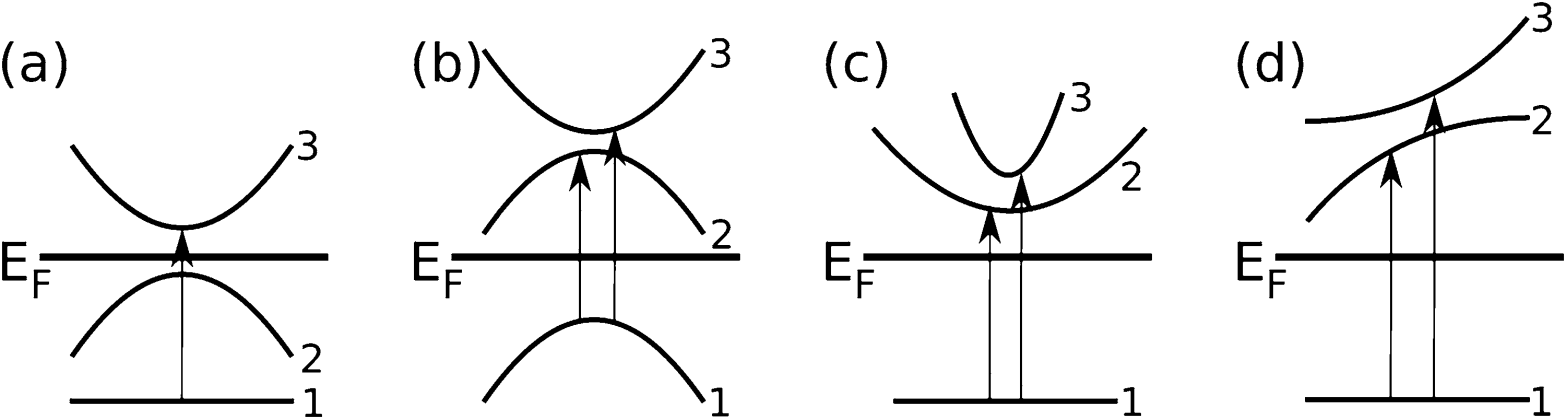
One dimensional sketches of possible MO transitions. In (**a**) transition 1 $\rightarrow$ 2 will not contribute to MO as both bands are occupied, thus transition 1 $\rightarrow$ 3 will not be weakened. This corresponds to the ideal case. In (**b**) the gradient of the energy difference (see Eq. (1)) causes transition 1 $\rightarrow$ 2 to be strong and narrow in energy and dominate over transition 1 $\rightarrow$ 3 which is weak and wide. This is the prime example of large JDOS discrepancy. In (**c**) and (**d**) we demonstrate that any assymetry in the hybridizing bands leads to non-zero total MO effect due to different JDOS.

The MO transitions have been classified by the dimensionality of the manifold that is formed by the hybridization of the generating bands as one- or two-dimensional, and by the position relative to the magnetization direction as parallel and perpendicular. The strongest signal to the MO spectra is provided by two-dimensional parallel transitions. We also present several possibilities of enhancing the MO signal (Fig. 9) by an appropriate band structure engineering.
